# Activity-Stability Relationships in Oxide Electrocatalysts for Water Electrolysis

**DOI:** 10.3389/fchem.2022.913419

**Published:** 2022-06-23

**Authors:** Marcus Wohlgemuth, Moritz L. Weber, Lisa Heymann, Christoph Baeumer, Felix Gunkel

**Affiliations:** ^1^ Peter Gruenberg Institute and JARA-FIT, Forschungszentrum Juelich GmbH, Jülich, Germany; ^2^ MESA+ Institute for Nanotechnology, Faculty of Science and Technology, University of Twente, Enschede, Netherlands

**Keywords:** water electrolysis, oxide electrocatalysis, activity-stability relations, perovskite—type oxide, green hydrogen, oxygen evolution reaction

## Abstract

The oxygen evolution reaction (OER) is one of the key kinetically limiting half reactions in electrochemical energy conversion. Model epitaxial catalysts have emerged as a platform to identify structure-function-relationships at the atomic level, a prerequisite to establish advanced catalyst design rules. Previous work identified an inverse relationship between activity and the stability of noble metal and oxide OER catalysts in both acidic and alkaline environments: The most active catalysts for the anodic OER are chemically unstable under reaction conditions leading to fast catalyst dissolution or amorphization, while the most stable catalysts lack sufficient activity. In this perspective, we discuss the role that epitaxial catalysts play in identifying this activity-stability-dilemma and introduce examples of how they can help overcome it. After a brief review of previously observed activity-stability-relationships, we will investigate the dependence of both activity and stability as a function of crystal facet. Our experiments reveal that the inverse relationship is not universal and does not hold for all perovskite oxides in the same manner. In fact, we find that facet-controlled epitaxial La_0.6_Sr_0.4_CoO_3-δ_ catalysts follow the inverse relationship, while for LaNiO_3-δ_, the (111) facet is both the most active and the most stable. In addition, we show that both activity and stability can be enhanced simultaneously by moving from La-rich to Ni-rich termination layers. These examples show that the previously observed inverse activity-stability-relationship can be overcome for select materials and through careful control of the atomic arrangement at the solid-liquid interface. This realization re-opens the search for active and stable catalysts for water electrolysis that are made from earth-abundant elements. At the same time, these results showcase that additional stabilization via material design strategies will be required to induce a general departure from inverse stability-activity relationships among the transition metal oxide catalysts to ultimately grant access to the full range of available oxides for OER catalysis.

## Introduction

Hydrogen is one of the most central and versatile chemical energy carriers envisioned as major ingredient for sustainable energy economies (Davis et al., 2018; Hauch et al., 2020; European Comission, 2020). Hydrogen provides high gravimetric energy density, is transportable, and can be used as reduction agent in chemical reactions or for conversion into electrical power via oxidation in fuel cell processes. Therefore, hydrogen-based processes may replace greenhouse gas emission-costly chemical production processes (e.g. in ammonia production, refining, steel and cement industry) and can compensate the intermittency of renewable energy sources, facilitating a globally interconnected CO_2_-neutral economy (International Energy Agency IEA, 2019).

**FIGURE 1:**
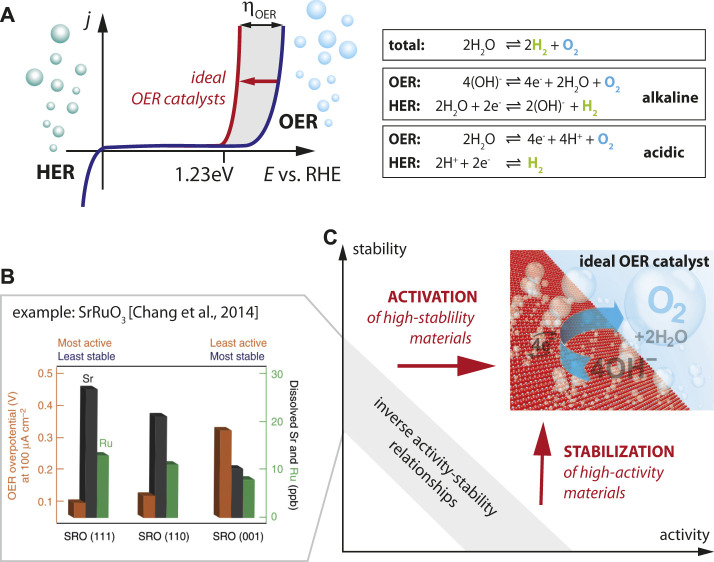
**(A)** The water splitting reaction in liquid electrolyte illustrating the two half-reactions of hydrogen evolution reaction and oxygen evolution reaction for alkaline and acidic media. Schematic behavior for ideal (red) and real (blue) OER catalysts. **(B)** Enhanced cation dissolution of Sr and Ru ions from SrRuO_3_ thin films for the most active crystal facets (111) [Reprinted by permission from Springer Nature, Nature Commun., Functional links between stability and reactivity of strontium ruthenate single crystals during oxygen evolution, Chang et al., 2014]. The inverse relation of chemical dissolution and required overpotential illustrates the inverse stability-activity relationship of standard OER catalysts. **(C)** Materials design strategies are needed to activate and stabilize OER catalysts in order to overcome the stability-activity dilemma.

A severe implication of this concept is the dire need for efficient production of hydrogen via CO_2_-neutral technologies. A key concept is the generation of such *green* hydrogen via water electrolysis, i.e. decomposition of water into the constituents (H_2_ and O_2_) by applying a potential (Figure 1A). This reaction proceeds in two half reactions, the hydrogen evolution reaction (HER) and the oxygen evolution reaction (OER). Water electrolysis arguably represents the simplest and most straight-forward process for the generation of green hydrogen, developed already decades ago (Bockris, 1947; Bockris, 1948; Hoare, 1969). The scientific and technological breakthrough, however, is still on hold due to fundamental challenges and inherent limitations in the “simple” process of splitting water: Catalysts are needed to provide suitable reactant adsorption sites, to stabilize the involved reaction intermediates, and to assist the implicit charge-transfer during OER. However, enhanced catalyst activity seemingly comes at the cost of a decreased stability and lifetime of the catalyst (Chang et al., 2014; Danilovic et al., 2014; Fabbri et al., 2014; Binninger et al., 2015; Fabbri et al., 2017; Yang et al., 2020), implying that good catalyst materials typically degrade very quickly, resulting in a rapid loss of the desired catalytic activity. This inverse behavior of stability and activity of catalyst materials involved in electrochemical processes presents a major obstacle for technology.

The underlying mechanisms and universality of this inverse stability-activity behavior are yet in discussion, and will be addressed in this perspective paper with a focus on OER catalysts. These reflect the main limitation of electrochemical efficacy due to significant overpotentials (*η*_OER_), reflecting the extra potential beyond the thermodynamic limit of 1.23 V vs. RHE (reversible hydrogen electrode) required to drive the water splitting reaction (Suntivich et al., 2011a; Hong et al., 2016; Vojvodic and Nørskov, 2011) in acidic as well as in alkaline water electrolysis, respectively (Figure 1A). Both scientific and technological advances for OER catalysts are urgently needed to arrive at realistic large-scale and energy-efficient application perspectives.

An additional point of consideration is the earth-abundance and supply risk of the catalyst materials. In acidic environment, noble metal catalysts can hardly be avoided as many non-noble transition metals and metal oxides are unstable in acid, resulting in the dissolution of such materials under working conditions. Acidic electrolysis technology therefore widely relies on platinum-group metal and metal oxide catalysts, such as IrO_*x*_, which raises the costs of this technology and at the same time limits the sustainability of such green hydrogen projects (Seitz et al., 2019). Conservative scenarios for hydrogen deployment in 2050 calculate that the roll-out of iridium based electrolyzers in Germany alone would require 37% of the annual world iridium production (Smolinka et al., 2018; Kiemel et al., 2021; Cherevko et al., 2016).

In contrast, many transition metal oxides and hydroxides are comparably stable in alkaline liquid environments, which allows using non-noble metal compounds to catalyze the OER in alkaline media (Suntivich et al., 2011a; Suntivich et al., 2011b). Typical benchmark materials are based on Ni, Co, Fe, including alloys and mixed oxide nanoparticles, such as NiFe and NiCo oxides, as well as oxy-hydroxide phases and spinels (Gong et al., 2013; Danilovic et al., 2014; Cherevko et al., 2016; Dionigi et al., 2020; Boucly et al., 2021). Moreover, perovskite oxides have been suggested as a versatile and highly active class of OER catalysts in alkaline media (Suntivich et al., 2011a; Grimaud et al., 2013), similarly with a focus on nickelates and cobaltates (Opitz et al., 2015; Gunkel et al., 2017; Cheng et al., 2018; Weber et al., 2019; Antipin and Risch, 2020; Yang et al., 2020; Baeumer et al., 2021a; Boucly et al., 2021). These two materials classes are near the top of the calculated volcano plot for perovskite oxides and thus provide the lowest required overpotentials among this class of materials (Suntivich et al., 2011a).

One of the major strengths of perovskite oxide catalysts is their flexibility in chemical, electronic and electrochemical properties (Risch, 2017; Gunkel et al., 2020a), owing to the intercoupled electronic-, orbital-, spin- and lattice (defect) degrees of freedom, which can be harvested for catalyzing electrochemical processes. For example, the choice of different transition metals allows tailoring the electronic structure and relative energy levels of catalyst and adsorbate electronic states as well as the mixed (oxygen-transition metal) orbital character and covalency of the employed electronic states (Vojvodic and Nørskov, 2011; Mefford et al., 2016; Montoya et al., 2017; Antipin and Risch, 2020). The growing ability to control perovskite oxides on the atomic scale is therefore promising to create a new boost for alkaline OER technologies through significant advances in controlling activity and stability of catalysts based on non-noble and earth-abundant metal oxide catalysts.

## Inverse Stability-Activity Relationships

Yet, major challenges remain: While general stability can be achieved in alkaline environment for oxide catalysts, it is still a big challenge to find both activity and stability in one and the same material, leading to the before mentioned inverse relationship between activity (determining electrochemical efficiency) and stability (determining catalyst lifetime). This behavior is typically rationalized by the fact that enhanced reaction rates (in the most active materials) at the same time lead to enhanced dynamics of undesired side or decomposition reactions, which take place in parallel to the OER and lead to a transient change of composition, structure, and catalytic behavior of the catalyst over time. These side reactions include (surface) phase changes, chemical leaching and dissolution of cations (Bick et al., 2016; Cherevko et al., 2016; Spoeri et al., 2017; Bick et al., 2018; Geiger et al., 2018; Weber et al., 2019) as well as potentially (un)desired reaction pathways, such as the evolution of oxygen from the catalyst lattice (lattice oxygen evolution reaction, LOER). LOER can take place in a non-destructive reversible manner (Grimaud et al., 2017), but can also facilitate a simultaneous dissolution of constituents (Fabbri et al., 2017). Therefore, the same composition that exhibits highest OER activity often shows an increase in undesired side reactions was mentioned in statement before already (Mefford et al., 2016), leading to fast degradation. For example, a strong transition metal 3*d*-oxygen 2*p* hybridization near the Fermi level can lead to higher OER activity, but also implies a mechanistic transition to a favored LOER (Mefford et al., 2016), which in turn decreases the catalyst lifetime and essentially results in the stability-activity dilemma (Fabbri et al., 2017).

For transition metal oxides, the inverse activity-stability relationship has been highlighted in the seminal paper by the *Markovic* group (cf. Figure 1B) which indicated the most rapid degradation of SrRuO_3_ catalysts for the most active crystallographic facets, illustrating the inverse correlation of OER activity and stability (Chang et al., 2014). Here, the degradation of the catalyst is parametrized by the amount of cations (i.e. Sr and Ru cations) dissolved from the catalyst into the electrolyte, which is most severe for (111)-oriented facets. Enhanced activity of the rapidly degrading catalysts is indicated by the lower overpotential required to drive the water splitting reaction (@100 μA/cm^2^) observed for the (111)-oriented sample. The behavior of SrRuO_3_ can hence be seen as characteristic for the inverse activity-stability relationship in OER catalysts, where a chemical dissolution process distorts and destabilizes the lattice, leading to a vanishing perovskite structure and eventually amorphization. A similar trend was also observed for pure metal catalysts (Danilovic et al., 2014; Lei et al., 2020).

This inverse behavior of stability and activity leads to the dilemma that typically materials can be chosen to be either “chemically stable” at “low activity” or to be “highly active” at “low stability”, while essentially both properties are needed in an ideal catalyst (Figure 1C). This *seems* to be a general trend, which is also confirmed in advanced SrRuO_3_-bilayer structures which succeeded to stabilize SrRuO_3_-based catalysts, but only at decreased activity. Here, SrRuO_3_ was buried under unit cell thick SrTiO_3_ capping layer that prevented excessive cation leaching (Akbashev et al., 2018).

## Atomistic Understanding of Activity and Degradation Relies on Atomically-Defined Sample Geometries

A key question in the field is what concepts can be applied to overcome this dilemma. This means that material engineering strategies are required that allow stabilizing the most active OER catalyst materials (horizontal arrow in Figure 1C) as well as for activating the most stable materials (vertical arrow in Figure 1C). This calls for decoupling the desired orbital and electronic degrees of freedom from chemical composition by material design to selectively suppress undesired side reactions such as chemical leaching, while maintaining high OER catalytic activity, and ultimately control activity and stability of the catalyst in an independent manner.

Through atomic-level control of synthesis in form of epitaxial thin films and heterostructures it is now possible to create desired combinations of different chemical compositions for perovskite oxides (Gunkel et al., 2017; Baniecki et al., 2019). This allows engineering surface cover layers (Akbashev et al., 2018; Heymann et al., 2022), and controlling chemical gradients and electronic properties independently via charge-transfer processes (Gunkel et al., 2020b; Burton et al., 2022) or sub-surface engineering (Akbashev et al., 2018; Zhang et al., 2020), and enables a systematic understanding and tuning of activity and degradation from atomically defined model systems (Weber and Gunkel, 2019). This enhanced material control with atomically smooth catalyst surfaces comes at the cost of a minimized contact area between catalyst and electrolyte, limiting the technological relevance of epitaxial systems. But such atomically controlled model systems have emerged as an ideal platform to identify structure-function-relationships at the atomic level, a prerequisite for advanced design rules (Risch et al., 2013; May et al., 2015; Scholz et al., 2016; Gunkel et al., 2017; Eom et al., 2018; Bak et al., 2019; Liu et al., 2019; Weber et al., 2019; Baeumer et al., 2021a; Baeumer, 2021; Baeumer et al., 2021b; Wan et al., 2021) and offering to finally overcome the materials challenge imposed by the activity-stability dilemma.

## Universality of Inverse Activity-Stability Relations—Examples of LaNiO_3-δ_ and La_0.6_Sr_0.4_CoO_3-δ_

Based on this approach, we address the question if inverse activity-stability relationships reflect a universal behavior of oxide OER catalysts or a material-specific behavior. For this, we compare LaNiO_3-δ_ (LNO) and La_0.6_Sr_0.4_CoO_3-δ_ (LSCO) epitaxial catalysts, which have both proven considerable OER activity in alkaline media (Weber et al., 2019; Baeumer et al., 2021a) and both group in close vicinity to ‘optimum’ catalyst behavior as predicted from theory (Suntivich et al., 2011a). Similar to Chang and co-workers (Chang et al., 2014), we use the crystallographic orientation of the active crystal facets as leading parameter to derive activity-stability relationships. The crystal orientation was controlled by means of oxide epitaxy via RHEED-controlled (reflection high-energy electron diffraction) pulsed laser deposition using SrTiO_3_ single crystalline substrates with defined surface orientations of (100), (110) and (111), respectively (cf. experimental section).

**FIGURE 2:**
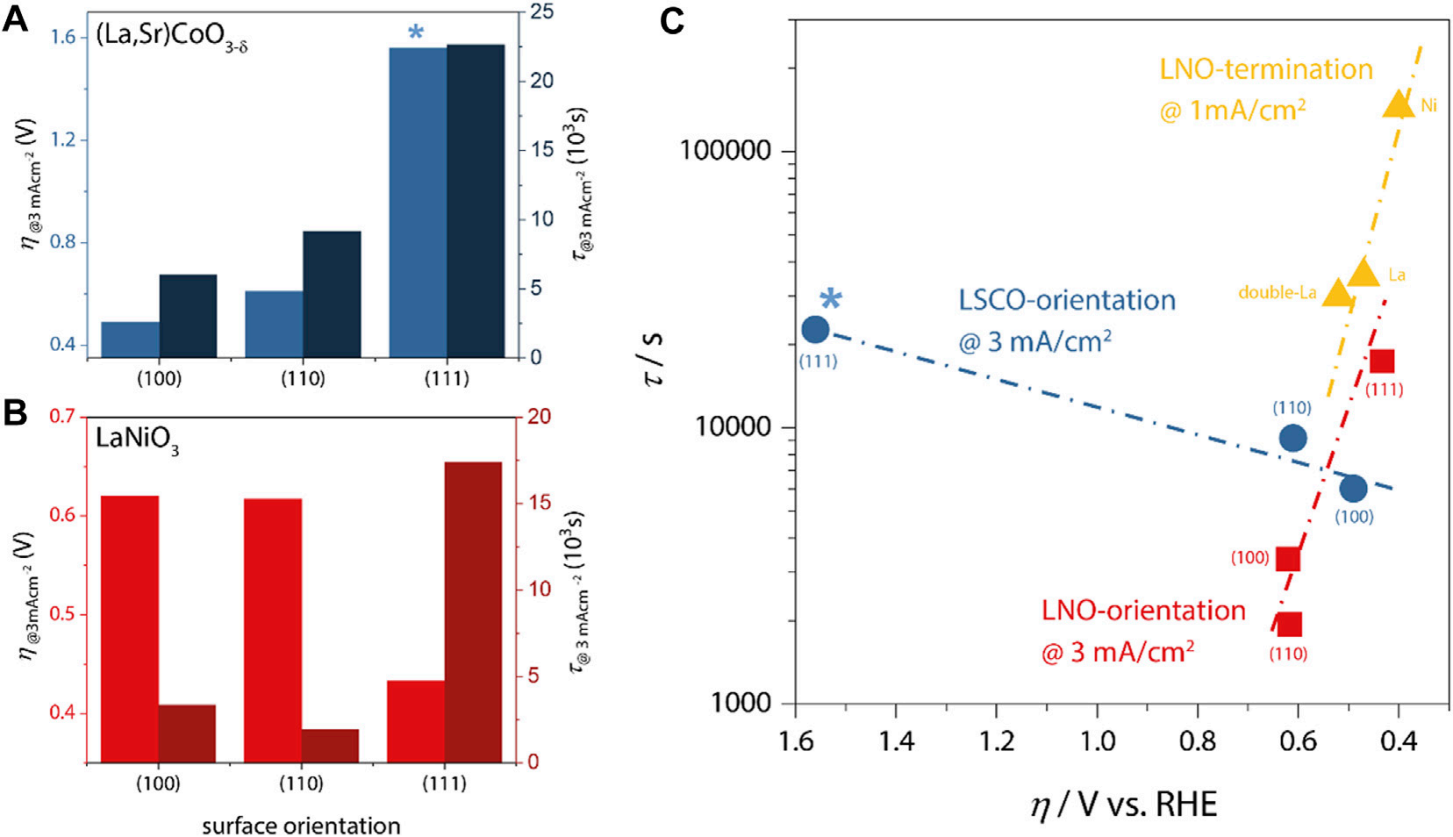
**(A,B)** Overpotential (left axes) and catalyst lifetime (right axes) at a load current density of 3 mA/cm^2^ for LSCO and LNO, respectively. *no IR-correction was applied for (111) LSCO because impedance spectra were inconclusive. The same qualitative trends are observed with and without IR-correction for all samples. **(C)** Lifetime vs. overpotential as a function of LSCO and LNO orientation (blue and red data, respectively) and LNO-termination [yellow; comparison of NiO_2_-termination (Ni), LaO-termination (La) and LaO-double-layer-termination (double-La)]. Note that termination-dependent measurements were performed at lower current density, which generally leads to higher lifetimes. Nevertheless, the same departure from inverse activity-stability trends is apparent for all LNO data.

In Figures 2A,B, we plot the lifetime and overpotential as derived from galvanostatic chronopotentiometry (CP) measurements for 20 nm thick LSCO catalysts and LNO catalysts, respectively. At a load current density of 3 mA/cm^2^, the epitaxial model catalysts typically remain active for 1,000 s to about 20,000 s. The lifetime was defined as the time interval from reaching the desired current density in CP measurements until a required potential of 3 V vs. RHE was observed. The corresponding overpotential was derived from the potential required to drive the desired current density during CP. Note that due to the atomically-smooth epitaxial character of these samples, the active surface area is identical to the geometric area exposed to the electrolyte, facilitating current normalization and allowing direct comparison of the collected data sets.

For LSCO, we observe the lowest overpotential of *η*_100_ ∼ 490 mV for the (100)-orientation, followed by (110)-orientation (*η*_110_ ∼ 610 mV) and finally the (111)-orientation with significantly increased *η*_111_ ∼ 1,560 mV. The corresponding lifetimes show the opposite trend, with the lowest value for (100)-orientation (*τ*_100_ ∼ 6,020 s) and increasing values for (110) and (111) (*τ*_110_ ∼ 9,160 s; *τ*_111_ ∼ 22,650 s). This inverse scaling behavior is summarized in Figure 2C relating the effective overpotential (on reversed axis) with the obtained lifetimes. Evidently, LSCO represents the typical inverse stability-activity relationship as was also found for SrRuO_3_ (Chang et al., 2014).

In contrast, LNO (Figure 2B) revealed both the longest lifetime and the lowest overpotential for the (111)-oriented sample (*η*_111_ ∼ 433 mV; *τ*_111_ ∼ 17,400 s), indicating a severe departure from the inverse activity-stability relationship (Figure 2C), as increased overpotential and intermediate lifetime are observed in (100)-orientation (*η*_100_ ∼ 620 mV; *τ*_100_ ∼ 3,350 s), and lowest lifetime at comparable activity is found for (110) (*η*_110_ ∼ 620 mV; *τ*_100_ ∼ 1,940 s). These data indicate that not only the intrinsic activity-stability relations differ between the two materials, but also that different crystallographic facets result in the highest OER activities despite the similar perovskite crystal structure of LNO and LSCO. To corroborate the distinct behavior of LNO, we additionally add lifetime-activity data (obtained @1 mA/cm^2^ resulting in generally higher lifetimes) determined for different atomic surface terminations, which for LNO can be obtained via control of the growth temperature [cf. Ref. (Baeumer et al., 2021a) for details]. The smallest overpotential and longest lifetime (*η*_Ni_1 mA/cm^2^∼400 mV; *τ*_Ni_1 mA/cm^2^∼144,000 s) is observed for Ni-rich terminations, with increasing overpotential and decreasing lifetime for increasingly La-rich surfaces (*η*_La_1 mA/cm^2^∼470 mV; *τ*_La_1 mA/cm^2^∼35,352 s; *η*_double-La_1 mA/cm^2^∼520 mV; *τ*_double-La_1 mA/cm^2^∼29,880 s), again confirming the systematic departure of the inverse relationship in LNO.

This systematic comparison of the structure-activity-stability relationship in LSCO and LNO hence principally proves that different catalyst materials may behave fundamentally different in terms of activity-stability relationships. In this particular case, LNO unites highest activity and stability in one specific surface termination and/or one specific orientation. In contrast, the classical inverse stability-activity relationship observed in LSCO showcases the necessity to derive suitable stabilization strategies that diminish the involved degradation processes, while maintaining its OER activity.

These examples demonstrate the importance of highly material-specific activity-stability relationships and that the previously observed inverse activity-stability-relationship can in fact be overcome for select materials and through careful control of the atomic arrangement at the solid-liquid interface. This realization re-opens the search for active and stable catalysts for water electrolysis that are made from earth-abundant elements. Inverse activity-stability relations are not necessarily universal and need to be checked individually for different materials and different leading parameters.

A remaining challenge in understanding this fundamentally different behavior is to differentiate between specific degradation behaviors under different electrochemical loads/conditions and the identification of the parameters that actually control the degradation of catalysts. For one, the degradation might be controlled electrochemically by the applied potential. Complementarily, degradation may be mediated by the surface-dynamics, i.e. the reaction rate at the active surface. For lower-activity samples, this implies that the CP (fixed current densities) will lead to higher required overpotentials as compared to higher-activity samples. At the same time, alternative stability test protocols, such as chronoamperometry (CA, fixed applied potential) will result in higher reaction rates and surface dynamics for more active samples. Therefore also the history of the applied current load protocols may play a role in determining comparable activity-lifetime data (Wei et al., 2019), highlighting the strong needs in the field to develop benchmark model systems and benchmark measurement protocols (Wei et al., 2019; Adiga and Stoerzinger, 2022). Especially, in the light of bringing the catalyst material to industrial application levels requires current load testing protocols that mimic grid fluctuations of different geographical regions. Additionally, real-time operando characterization of electrochemical interfaces are necessary to further track the complex interplay of surface-dynamics, electrochemical potential, and degradation behavior (Baeumer, 2021).

## Discussion and Perspective

In this perspective, we discussed the material design of non-precious metal oxide OER catalysts as a highly active and dynamic field. Current efforts are driven by the fact that besides activity the electrochemical stability of OER catalysts became a main limiting factor for the application of many transition metal oxide catalysts in water splitting applications. It is highly needed to systematically break the oftentimes observed inverse stability-activity relationships and to find appropriate materials and material combinations that depart from this undesired limitation. As it turns out, this can be achieved by systematically identifying select materials such as shown for the example of LNO. For LNO, proper selection of surface termination and crystal orientation breaks the activity-stability dilemma whereas the observed activity and stability for LSCO’s different crystal orientations do not break this dilemma.

Revealing the mechanistic nature behind this non-universal activity-stability behaviors observed for perovskite oxide OER catalysts requires a detailed understanding of the atomistic processes during OER and in particular a detailed understanding of the specific degradation mechanisms. The non-universal activity-stability behavior may be influenced by various parameters, such as different thermodynamically preferred and kinetically limited surface compositions, a differing defect chemistry or element solubility in the adjacent electrolyte. As a general theme, tuning the total energy of the catalyst by materials engineering can provide an interesting concept to stabilize those materials which suffer from the inverse stability-activity relationship. We hypothesize that the development of specific advanced material combinations may overcome the activity-stability dilemma when single compound catalysts follow the inverse behavior such as in the cases of LSCO or SrRuO_3_. Epitaxy of complex oxides can provide model systems to enhance the systematic understanding of activity and degradation, and enables tailoring and creating new material combinations with hybrid properties and dedicated electronic and chemical structures, therefore yielding a huge opportunity towards novel catalyst design.

Such advanced material designs give broad opportunities to improve the stability of perovskite oxide catalysts such as combining current collectors, active and/or stable catalyst materials in multi-layer structures (Akbashev et al., 2018; Baniecki et al., 2019), tuning surface stoichiometry (Baeumer et al., 2021a), applying modulation doping (Heymann et al., 2022), chemical substitution and atomic ordering phase-control (Gunkel et al., 2017; Zhu et al., 2021; She et al., 2022). In addition to material optimization, electrolyte engineering can be applied to avoid surface passivation (Chung et al., 2020).

Nevertheless, further atomistic understanding is required more than ever to distinguish the chemical nature of inverse activity-stability-type catalysts and to provide the required guidelines to overcome the current stability limitations of OER catalysts. This is even more emphasized when considering further challenges in the field beyond activity-stability-relations; single compound catalysts comply with intrinsic scaling relations (Man et al., 2011), which describe the inherent correlation of the energy barriers of the four-step reaction intermediates during OER, and limit the ability to control the adsorption energies of intermediate reaction steps individually. Here, catalysts with “hybrid-material” properties achieved through nano-scale engineering of composition and structure may provide optimum parameters for select reaction intermediates, allowing to further decrease overpotential while maintaining catalyst lifetimes and stability.

In addition, the establishment of catalyst design rules is complicated by chemical surface transformations taking place under OER conditions. These were revealed for various oxide OER catalysts including the class of perovskite oxides and imply the occurrence of (surface) phase transition of the catalysts when electrochemical bias is applied. This poses an additional challenge towards understanding the atomistic processes during the OER and the relevant processes leading to degradation, and towards revealing the holistic aspects underlying the inverse stability-activity relationships, scaling relations, chemical transformations and their dependence on the applied potential and aging protocols.

Therefore, OER catalyst research and material design remain rich fields for science and technology with the potential of providing groundbreaking solutions towards efficient and sustainable hydrogen-based energy concepts, especially when combining novel nanoscale materials engineering and operando or *in situ* characterization of the composition, structure, and electronic properties. These efforts should focus on noble metal free electrode materials to achieve a long term sustainable hydrogen economy as resources are preserved and application costs are reduced. We showed that the activity-stability dilemma for perovskite oxide OER catalysts can be overcome and discussed various design options for improvement of the catalytic activity as well as the stability.

## Experimental

### Epitaxy of LSCO Thin Films

Epitaxial 20 nm thick La _0.6_Sr_0.4_CoO_3-δ_ thin films were deposited on epipolished single-crystalline (001), (110) and (111) SrTiO_3_ substrates (Shinkosha Co. Ltd.) by reflection high-energy electron diffraction (RHEED)-controlled pulsed laser deposition (PLD, Twente Solid State Technology). The PLD was operated with a KrF-excimer laser (Lambda Physik Lasertechnik) with a wavelength of *λ* = 248 nm using a repetition rate of *f* = 5 Hz and a laser fluence of *F* = 2.19 J/cm^2^. The substrate temperature was *T* = 650°C and the target-to-substrate distance was 60 mm. The oxygen partial pressure was *p* (O_2_) = 0.053 mbar.

### Epitaxy of LNO Thin Films

Epitaxial 20 nm thick LaNiO_3-δ_ thin films were likewise deposited by RHEED-controlled PLD (SURFACE systems + technology GmbH), using a repetition rate of *f* = 5 Hz and a laser fluence of *F* = 1.60 J/cm^2^ at a substrate temperature of *T* = 550°C and *p*(O_2_) = 0.020 mbar. After deposition, the LNO/STO samples were annealed at *p*(O_2_) = 0.1 mbar for 10 min and subsequently quenched. For termination control, the growth temperature was systematically varied between *T* = 550 and 800°C [cf. Ref. (Baeumer et al., 2021a)].

### Electrochemical Lifetime and Activity Measurements

The catalyst activity and lifetimes were characterized using a rotating disc electrode setup (Pine Research) with a custom-made adapter for the thin film electrodes and a potentiostat (Bio-Logic Science Instruments). Before the measurements, 50 nm thick Pt contacts were sputtered at the edges of the thin films as well as the backside of the sample in order to ensure sufficient electronic contact to the Pt stamp of the RDE shaft. On the front side, a film area of 0.75 mm diameter was exposed to the electrolyte and sealed using an O-ring (Kalrez, ERIKS, Germany). A rotation rate of 1,600 rpm was applied and O_2_-saturated 0.1 M KOH (obtained from KOH pellets, Sigma Aldrich, 99.99%) was used as electrolyte. The measurements were performed at room temperature in a 150-ml alkaline-resistant Teflon cell (Pine Research) with a Pt coil reference electrode. Potentials were referenced to a Hg/HgO reference electrode (CHI Instruments, United States), which was periodically calibrated to the reversible hydrogen electrode (HydroFlex, United States) in 0.1 M KOH with typical values of ∼890 mV. Electrochemical impedance spectroscopy (EIS) was applied to correct for the uncompensated series resistance *R*_*s*_ of the catalysts (*iR*-correction), while the lifetimes were tested by chronopotentiometry (CP). During CP, the current density was initially ramped up in a quasi-static staircase measurement [cf. Ref. (Heymann et al., 2022)], before keeping the current density at 1 mA/cm^2^ and 3 mA/cm^2^, respectively.

## Data Availability

The original contributions presented in the study are included in the article/supplementary material, further inquiries can be directed to the corresponding authors.
